# Income-related inequality and decomposition of edentulism among aged people in China

**DOI:** 10.1186/s12903-022-02246-7

**Published:** 2022-05-31

**Authors:** Shuo Du, Menglin Cheng, Chunzi Zhang, Mengru Xu, Sisi Wang, Wenhui Wang, Xing Wang, Xiping Feng, Baojun Tai, Deyu Hu, Huancai Lin, Bo Wang, Chunxiao Wang, Shuguo Zheng, Xuenan Liu, Wensheng Rong, Weijian Wang, Tao Xu, Yan Si

**Affiliations:** 1 Department of Preventive Dentistry, National Engineering Laboratory for Digital and Material Technology of Stomatology, Beijing Key Laboratory of Digital Stomatology, National Clinical Research Center for Oral Diseases, Research Center of Engineering and Technology for Digital Dentistry of Ministry of Health, Peking University School and Hospital of Stomatology, Beijing, 100081 China; 2 Department of Stomatology, Beijing Friendship Hospital, Capital Medical University, Beijing, China; 3 Distinct Health Care, Chengdu, China; 4 Department of Stomatology, Beijing Chao-Yang Hospital, Capital Medical University, Beijing, China; 5 Chinese Stomatological Association, Beijing, China; 6 Shanghai Ninth People’s Hospital, Shanghai Jiao Tong University School of Medicine, Shanghai, China; 7 School and Hospital of Stomatology, Wuhan University, Wuhan, China; 8 West China School of Stomatology, Sichuan University, Chengdu, China; 9 Guanghua School of Stomatology, Hospital of Stomatology, Sun Yat-Sen University, Guangzhou, China; 10 Chinese Center for Disease Control and Prevention, Beijing, China

**Keywords:** Edentulism, The elderly, Inequality, China

## Abstract

**Background:**

The aim of this study was to assess the income-related inequality of edentulism among the aged in China and identify the contributing factors.

**Methods:**

A secondary analysis of data from the 4th National Oral Health Epidemiology Survey in China was conducted, and 65–74 years old were selected for the analysis of income-related inequality of edentulism. The concentration curve, Concentration index (CI) and Erreygers-corrected concentration index (EI) were used to represent inequality and its degree qualitatively and quantitatively, respectively. A decomposition method based on probit model was employed to determine the contributors of inequality, including demographic factors, income status, oral health-related knowledge, attitude and practices and self-perceived general health status.

**Results:**

In China, aged people with edentulism were concentrated in the poor. The CI was − 0.2337 (95% CIs: − 0.3503, − 0.1170). The EI was − 0.0413 (95% CIs: − 0.0619, − 0.0207). The decomposition results showed that income (75.02%) and oral health-related knowledge, attitude and practices (15.52%) were the main contributors to the inequality.

**Conclusion:**

This study showed that pro-poor inequality among the elderly with edentulism existed in China. Corresponding policies against the contributors could be considered to promote the health equality of the elders.

**Supplementary Information:**

The online version contains supplementary material available at 10.1186/s12903-022-02246-7.

## Background

Edentulism, the complete loss of all natural teeth, is considered a cumulative and end result of dental diseases [1]. Among the edentulous, the nutrition will be compromised since they cannot chew efficiently [2]. Speaking unclearly and facial sagging may affect communication and even produce inferiority, compromising the quality of life [3–6]. Furthermore, edentulism has also been associated with general health such as cardiovascular diseases and cognitive impairment [7–9]. Edentulism and severe tooth loss remains a large oral disorder disability burden globally [10]. As an important and comprehensive indicator of oral health, especially for the elderly, tooth loss is monitored globally as an effective marker of population oral health [11].

Against the aging of the population, there will be 400 million people aged 65 and older by 2050 in China [12]. Despite the situation of edentulism among the elderly in China has improved compared with 10 years ago [13], we are still faced with great challenges for medical care of the elderly. Studies have shown the significant association between edentulism and socio-demographic characteristics [14–16], health behaviors [14, 15, 17, 18] and self-rated general health [19, 20]. Furthermore, socioeconomic status is also significantly associated with oral health behaviors [21–23].

Health equality refers to that the distribution of health should be need-oriented rather than depends on social privileges [24]. However, socioeconomic gradient in edentulism has been found in developed and developing countries [15, 25–27], but there exist scant researches on measuring and decomposing socioeconomic inequality related to edentulism globally.

The concentration curve and the concentration index (CI) are one of the most common ways to measure inequalities and decomposition method helps to monitor and understand the determinants of inequality [28]. Using the data from the 4th National Oral Health Epidemiology Survey (NOHES) conducted in 2015–2016, the purpose of this study was to measure the income-related inequality of edentulism among aged people in China and then decompose socioeconomic inequality in edentulism to determine the relevant contributing factors providing basis for formulating corresponding policies.

## Methods

### Data

The 4th NOHES was a cross-sectional study covering 31 provinces, autonomous regions and municipalities adopting a multistage, stratified, equal volume and random sampling method. Oral health examinations and oral health-related questionnaires were conducted according to the standards of World Health Organization (WHO) [29]. The specific research methods have been described in detail in the previous study [30]. The study protocol was approved by the Stomatological Ethics Committee of the Chinese Stomatological Association (No. 2014-003).

The data of 65–74 years old (N = 4431) were selected for analysis. A sample of 3463 participants remained after exclusions of those with missing or abnormal values of household income (n = 965) and missing values of family scale (n = 3). Based on the data from the 6th National Census in 2010 [31], the samples were post-stratified by province, area (urban or rural) and gender. The standard survey weights were then calculated to obtain an unbiased estimate of the overall population [32, 33].

### Variables

A binary outcome variable- edentulism was created based on the oral examination (0:number of natural teeth remaining > 0; 1:number of teeth remaining = 0). As per previous studies [14–20], determinants of edentulism included demographic factors (gender, area and educational attainment), annual household income per capita, oral health knowledge, attitude and practice (oral health knowledge, attitude, smoking status, dental service utilization and medical insurance) and self-perceived general health. Educational attainment levels were divided into 3 groups: low group (junior high school and below), medium group (medium-technical secondary school and senior high school) and high group (junior college and above). Annual household income per capita were also categorized into quintiles according to the sample frequency. Oral health related attitude was define as negative (correct answers < 3 questions in 4) or positive (correct answers ≥ 3 questions in 4); Oral health related knowledge was defined as low (correct answers < 5 questions in 8) or high (correct answers ≥ 5 questions in 8).

### Measuring inequality

The concentration curve and concentration index (CI) [34] were used to measure the degree of income-related inequality in edentulism among the elderly.

First, the participants were ranked according to annual household income per capita, and the prevalence of edentulism was calculated. Then, the relationship between the prevalence of edentulism and income level was preliminarily analyzed by Chi-square test. On this basis, a concentration curve was formed by plotting the cumulative proportion of edentulism in the population (Y-axis) against the cumulative population (X-axis) ranked by socioeconomic factors from the poorest to the richest. If the distribution of edentulism was perfectly equal among individuals, the result would be a 45-degree line (the equality line). If there existed inequality, the concentration curve would lie between the X- and the Y-axis and the equality line. The CI was equal to 2 times the area between the concentration curve and the equality line. The inequality of edentulism was pro-poor when the index was negative, and the concentration curve was above the equality line. The positive index indicated pro-rich inequality with the concentration curve below the equality line. CI is defined as follows:

$$CI = \frac{2}{{\overline{y}}}cov\left( {y,\;r} \right)$$

where $\overline{y }$ is the mean of the health outcome variables- edentulism, and *r* is the fractional rank of individuals in the personal income distribution.

However, Erreygers [35] pointed out that when the outcome variable was binary, the value of the index was arbitrary to a large extent and proposed to solve this issue by adjusting the original index using the mean, minimum and maximum value of the outcome variable. Considering that, Erreygers-corrected concentration index (EI) is preferred to measure inequality of edentulism. And the natural log of the annual household income per capita was used to calculate EI. EI can be written as follow:

$$EI = \frac{{4\overline{y}}}{{y_{max} - y_{min} }}\;CI$$

where $y_{max}$ and $y_{min}$ represent the upper and lower limits of edentulism respectively. Therefore, it can be simplified as:

$$EI = 8cov\left( {y,\;r} \right)$$

where *y* is the health outcome variables- edentulism, and *r* is the fractional rank of individuals in the personal income distribution.

### Decomposing inequality

Decomposition analysis examines the contribution of personal factors to income-related inequality among the elderly [36]. A probit model was used for the decomposition of the EI and factors associated with the inequality of edentulism were mainly classified into 4 sources: (i) demographic factors, (ii) income, (iii) oral health knowledge, attitude and practice and (iv) self-perceived general health status. Each contribution shows how much variation of the determinant across income level can explain the observed association between income and edentulism. [36]. Positive contribution rate indicates that the variable would add the inequality in health variable and vice versa. For decomposition, EI can be expressed as follow:

$$EI = 4*\left( {\mathop \sum \limits_{k} \left( {\beta_{k}^{m} \overline{x}_{k} } \right)C_{k} + GC_{\varepsilon } /\overline{y}} \right)$$

where $\overline{x}_{k}$ is the mean of the determinants included in the decomposition analysis, $\beta_{k}^{m}$ is the marginal effect evaluated at the sample means, $C_{k}$ is the concentration index of the determinants, and $GC_{\varepsilon }$ is the generalized concentration index of the error term $\varepsilon$.

Sensitivity analysis was conducted to assess the robustness of the decomposition model by changing reference groups [37].

All statistical analyses were conducted by STATA 15 (STATA Corporation, College Station, TX).

## Results

Table 1 illustrates the descriptive characteristics of people sampled into the survey. The prevalence of edentulism was 4.42% (95% CIs: 3.36–5.78%).

**Table 1:** Descriptive characteristics of participants

Variables	Poststratification data	
	Proportion (%)	95% CIs
*Edentulism*		
No	95.58	(94.22, 96.64)
Yes	4.42	(3.36, 5.78)
*Gender*		
Male	49.93	(49.04, 50.83)
Female	50.07	(49.17, 50.96)
*Area*		
Urban	42.92	(36.69, 49.39)
Rural	57.08	(50.61, 63.31)
*Educational attainment level*		
Low	84.72	(81.69, 87.33)
Medium	10.67	(9.00, 12.59)
High	4.61	(3.30, 6.40)
*Income*		
1st quintile (poorest)	21.87	(17.42, 27.08)
2nd quintile	18.99	(15.92, 22.50)
3rd quintile	20.28	(17.77, 23.05)
4th quintile	20.74	(17.94, 23.84)
5th quintile (richest)	18.12	(14.66, 22.19)
*Smoking*		
Yes	25.54	(23.55, 27.64)
Never	61.18	(59.40, 62.94)
Quit smoking	13.28	(12.00, 14.67)
*Perceived general health*		
Very poor/poor	18.80	(16.17, 21.76)
Fair	44.27	(41.21, 47.38)
Good/ very good	36.93	(33.54, 40.45)
*Dental service utilization in the past 12 months*		
Yes	20.81	(18.7, 23.10)
Never	79.19	(76.90, 81.30)
*Attitude*		
Low	22.32	(18.82, 26.25)
High	77.68	(73.75, 81.18)
*Knowledge*		
Low	54.52	(49.85, 59.11)
High	45.48	(40.89, 50.15)
*Medical insurance*		
No	1.82	(1.25, 2.64)
Yes	98.18	(97.36, 98.75)

**Fig. 1:**
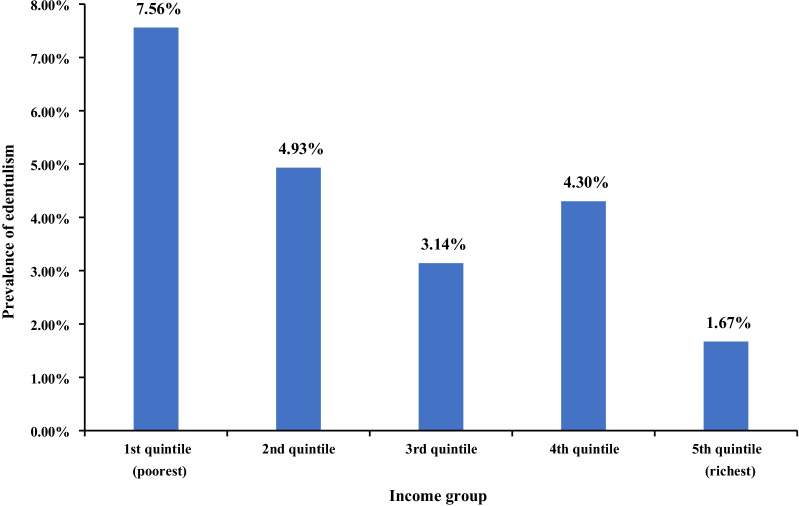
Distribution of edentulism by income among the elderly aged 65–74 in 2015 (Chi-square test was applied. *P* $<$ 0.01)

Figure 1 shows a clear socioeconomic gradient for the prevalence of edentulism by income among people aged 65–74 in 2015 (*P* $<$ 0.01).

**Fig. 2:**
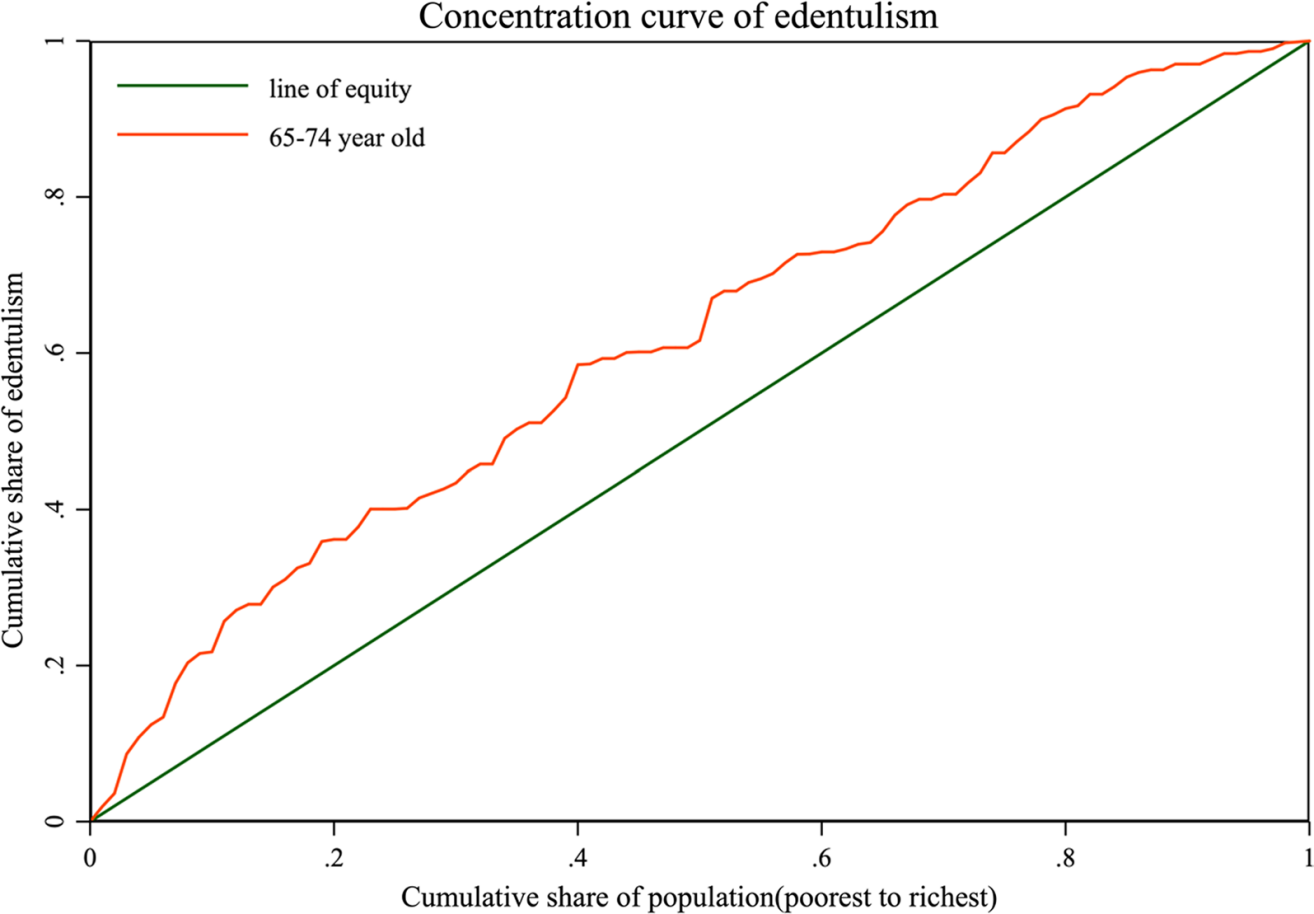
Concentration curve for edentulism among the elderly in 2015

The concentration curve shown in Fig. 2 was above the equality line, indicating that edentulism is concentrated mainly in the poor. Table 2 exhibits that the CI and EI of edentulism among the elderly (65–74 year-olds) were − 0.2337(95% CI: − 0.3503, − 0.1170) and − 0.0413(95% CI:  − 0.0619, 0.0207) respectively. Both CI and EI are negative, which were consistent with concentration curve.

**Table 2:** Concentration indices for edentulism by income among the elderly in China in 2015 (N = 3463)

Status of tooth loss	CI	EI
Edentulism 95% CIs	− 0.2337^***^ (− 0.3503, − 0.1170)	− 0.0413^***^ (− 0.0619, 0.0207)

***P < 0.001

Table 3 presents the decomposition of inequality in edentulism by income among the elderly. Undoubtedly income (75.02%) was a major contributor to the inequality of edentulism for the elderly. The second-largest contributor was oral health attitude, knowledge and practices (15.52%). All behaviors beneficial to oral health (never/quit smoking, visiting the dentist in the past 12 months, positive attitude, high knowledge and having medical insurance) were concentrated in the rich. Demographic characteristics (11.61%) and self-rated general health status ( − 6.16%) did not contribute much to the results. However, the latter one had a negative contribution to the inequality, suggesting it reducing the inequality of edentulism. Results were robust in the sensitivity analysis (see Additional file 1).

**Table 3:** Decomposition of Inequality in lack of functional dentition by income among the elderly in China in 2015 (N = 3463)

Variables	Elasticity	Concentration Indices	Contribution	%
**Demographic variables**				**11.61**
*Gender*				
Male	Reference			
Female	0.0615	− 0.0057	− 0.0003	0.84
*Area*				
Urban	Reference			
Rural	0.0192	− 0.0925	− 0.0018	4.31
*Educational attainment level*				6.46
Low	Reference			
Medium	− 0.0005	0.3944	− 0.0002	0.46
High	− 0.0036	0.6968	− 0.0025	6.00
**Income**^#^	− 0.4664	0.0664	− 0.0310	**75.02**
**Knowledge, attitude and practices**				**15.52**
*Smoking*				1.96
Yes	Reference			
Never	− 0.0471	0.0212	− 0.0010	2.42
Quit smoking	0.0023	0.0827	0.0002	− 0.46
*Dental service utilization in the past 12 months*				
No	Reference			
Yes	− 0.0095	0.2017	− 0.0019	4.62
*Attitude*				
Negative	Reference			
Positive	− 0.0725	0.0254	− 0.0018	4.46
*Knowledge*				
Low	Reference			
High	− 0.0371	0.0508	− 0.0019	4.56
*Medical insurance*				
No	Reference			
Yes	0.0505	0.0007	< 0.0001	− 0.08
**Perceived general health**				− **6.16**
Very poor/poor	Reference			
Fair	0.0250	0.0040	0.0001	− 0.24
Good/very good	0.0266	0.0919	0.0024	− 5.92
**Residual**			− 0.0017	**4.01**

^#^Natural log of annul household income per capita was calculated in the regression

Primary categories of contributing facotrs are in bold and aggregated contribution of each primary category is in bold

## Discussion

In recent years, numerous studies about the socioeconomic-related inequality of oral disease sprang up, disclosing the phenomenon that the marginalized people suffer from the oral disease disproportionally [38–40]. This study is the first to comprehensively use data from the 4th NOHES to evaluate and decompose the socioeconomic inequality of edentulism among Chinese elders mainly measuring such inequality related to income.

The prevalence of edentulism among the participants is relatively low compared to other countries [25, 26, 41]. Given the low oral health care utilization over adult in China [22], it is speculated to be related to the situation that the residual crown or roots not worth preserving remained unextracted. As the positive association between age and edentulism [14], the age range of different studies should also be considered.

Socioeconomic condition is a broad concept including income, occupation, level of education and so on [42]. Considering the age of participants (65–74 years old) and the year of the investigation (2015), most of them were born in 1950s. They spent childhood and the early adulthood before the issue of the Compulsory Education Law in 1986. As a result, 84.72% of the elders were with junior high school or below. Therefore, income was chosen to measure the socioeconomic condition in this study.

The EI with negative value indicated that there was pro-poor inequality in edentulism among the elderly. The socioeconomic gradient observed in this study was consistent with previous studies [1, 16, 26, 27], although there was considerable variation in the magnitude of inequalities in edentulism across these countries. It might be due to the difference of analysis approaches, age groups and extent of income inequality in the studies.

The decomposition of the result revealed that income made great contributions to the pro-poor inequality in edentulism among the elderly, which is in consistency with previous studies [1]. Also, Seerig et al. analyzed the relationship between income and tooth loss in 11 studies and demonstrated that lower level of income presented a greater chance of tooth loss [42]. Previous studies have indicated possible explanations. First, income inequality may lead to the reduction of investment on the public health resources such as water fluoridation [42, 43]. Secondly, the percentage of routine expenditure on health care is low among the low-income group and the dental expenditure is still a tiny fraction in terms of the health care payments in China [44]. Besides, studies showed that people with low incomes tend to neglect health-related behaviors such as smoking and regular health care [21, 22, 45]. Based on the 4th NOHES in China, we found out that dental service utilization disproportionately concentrated in better-off adults [22]. In addition, financial constraints are closely related to the type of dental treatment provided. Lower-income group may prefer tooth extraction, while higher-income group tend to regular dental appointments and conservative dental treatment with a greater number of retained teeth [46, 47]. Also, low-income population usually suffer more stress in life. Tooth loss may be influenced by “stress-induced oral-health-related behaviors” and psychological effects, which may be related to tooth retention [48]. Generally speaking, inaccessible dental care, unaffordable dental cost and treatment-focused approach may be account for the inequality especially for low income group. Therefore, oral health care system may shift to a teamwork to meet needs of all population and be integrated with primary health care system to decrease health inequality.

Oral health knowledge, attitude and practice were also important factors for the inequality. Oral health literacy is closely related to an individual’s capacity to obtain, process, interpret or understand basic oral health information and make appropriate decisions on oral health care promotion, prevention, and utilization. It has been found to play a mediating effect between socioeconomic factors and tooth loss. Oral health literacy not only affect tooth loss directly, but also have an indirect influence on tooth loss through oral health practices. People with low oral health literacy are more likely to brush teeth less, go to the dentist irregularly and have less preventive consultation, which may result in worse oral health outcomes [18]. Efforts should be made to improve oral health literacy to reduce oral health disparity and promote oral health. Oral health practices are also influenced by the social environment- a complex of family/household, peers, class, neighborhood, workplace, ethnicity, societal structure and so on [49]. Under a behavioral ecology perspective, Nettle put forward a model that for people with low socioeconomic status, the lower investment in healthy behaviors is the adaptive adjustment according to their own situation [50]. Therefore, for the poor, primary oral health care based on legislative or economic incentives may be effective in reducing health inequality.

Though the educational level did not make much contribution to the inequality of edentulism in the study, studies have disclosed the association between education and tooth loss [16, 26, 51]. And Yusuke Matsuyama et al. provided causal evidence on the effects of education on oral health outcomes including edentulism [52]. Education may not only have an impact on health due to its impact on improving health-related behaviors throughout adult life directly [53, 54], but also have an indirect effect by providing better employment opportunities, higher income and better living conditions. Thus, investing more resources to develop the coverage rate for compulsory education is essential.

In our study, good self-rated general health and dentate group were more prevalent among the higher income population and self-rated general health reduced the inequality of edentulism. Studies suggest that there is a negative association between the prevalence of edentulism and self-reported general health [19, 20]. Poor general health have been found to be associated with more dental visit [55, 56]. Therefore, poor self-experienced overall health may have a positive effect on tooth remaining for low-income population by improving dental care utilization among them, which suggests that we should focus on the prevention and control of general health.

The rest of the inequality was the residual component of the EI, implying that there was some inequality in edentulism which could not be explained by the determinants mentioned above.

The advantages of this study are as follows: first, it used national data from the 4th NOHES in China, which covered a wide range of areas adopting international advanced standards in terms of its contents, methods and standards. Second, research in this field in China is limited, and this study presents essential evidence of inequality in edentulism among the aged population in China providing a basis for future research. Finally, our study used concentration curves, CI and EI to measure the degree of income-related inequality in edentulism among the elderly and then established a decomposition model to identify relevant factors contributed to this inequality, providing related theoretical basis for government policy-making.

Some limitations of this study must be considered. First, the findings were based on cross-sectional data, so the causal relationship cannot be determined between edentulism and associated variables in the elderly. Therefore, these findings should be interpreted cautiously. Second, the data of the questionnaire were derived from self-reports. Thus, recall bias may cause inaccurate results. In addition, socioeconomic factors other than income (e.g. education, wealth and occupation) were not assessed. Finally, with the increase of life expectancy, the average life expectancy of the Chinese population has exceeded 76 years old in 2015[57]. However, the participants of this study only included the elderly aged 65–74 years. In the future, we could expand the age range to study the socioeconomic inequality of edentulism among the elderly more comprehensively. Despite the limitations, our results indicate a relationship between income and edentulism among the aged in China. Further investigations with a longitudinal design and broader socioeconomic status measurements adopting life course approach are encouraged to confirm our findings.

## Conclusions

In summary, this study proved that there is a pro-poor inequality in edentulism among the elderly in China. Income status and oral health knowledge, attitude and practice are the main factors contributing to this inequality. This study offers policymakers information on contributing factors of inequalities on edentulism in the elderly in China. Dental care systems should focus more on promoting and maintaining oral health to achieve greater oral health equality.

## Supplementary Information

**Additional file 1: Table S1.** Decomposition results, changing the reference category in the probit regression model.

## Data Availability

The data that support the findings of this study are available from the National Health Commission of the People's Republic of China. Restrictions apply to the availability of these data, which were used under license for this study. Data are available from the authors with the permission of the National Health Commission of the People's Republic of China.

## Abbreviations

NOHES: National Oral Health Epidemiology Survey
WHO: World Health Organization
CI: Concentration index
EI: Erreygers-corrected concentration index
CIs: Confidence intervals
