# Supplementary material for: Income-related inequality and decomposition of edentulism among aged people in China
Source: BMC Oral Health. 2022 May 31;22:215. doi: 10.1186/s12903-022-02246-7 (PMC9153164; doi:10.1186/s12903-022-02246-7)
Supplement: Supplementary file 1 — Additional file 1: Table S1. Decomposition results, changing the reference category in the probit regression model. [file 12903_2022_2246_MOESM1_ESM.docx]

**Additional Table. Sensitivity analysis (changing the reference category).**

| **Variables** | **Contribution %** |
| --- | --- |
| **Demographic variables** | **8.44%** |
| **Income^#^** | **75.02%** |
| **Knowledge, attitude and practices** | **15.97%** |
| **Perceived oral health status** | **-5.25%** |

^#^Natural log of annul household income per capita was calculated in the regression.
